# Determination of the stability of sodium cyclamate during deep-frying using HPLC

**DOI:** 10.1371/journal.pone.0308220

**Published:** 2024-08-08

**Authors:** Li Mu, Yuhang Liu, Huihong Luo, Qianqian Liu, Li Zhang, Ying Xu, Gang Li, Qi Tong

**Affiliations:** 1 College of Food Science and Engineering of ChangChun University, Changchun, Jilin, China; 2 Jilin Province Product Quality Supervision and Inspection Institute, Changchun, Jilin, China; 3 Pony Testing International Group (Tianjin), Tianjin, China; University of New Hampshire, UNITED STATES OF AMERICA

## Abstract

The oil used to fry food is often used multiple times to reduce costs. However, when foods containing sweeteners are processed in this way, the sweeteners may produce substances harmful to the body as a result of repeated frying at high temperatures. This article investigated the stability of sodium cyclamate during deep-frying by HPLC using a pre-column derivatization method. The results showed that cyclohexylamine was a decomposition product of a standard sample of sodium cyclamate when deep-fried at 200°C for 25 min. A pre-column derivatization/HPLC method was established to determine cyclohexylamine, a decomposition product of sodium cyclamate, under these conditions. Dansyl chloride was used as the derivatization reagent, the derivatization temperature was 60°C, the derivatization time was 20 min, the pH of sodium bicarbonate buffer solution was 11, and the concentration of dansyl chloride was 2.0 mg/mL. Detection was carried out by using an Agilent 1260 high-performance liquid chromatograph coupled with an ultraviolet detector. The ultraviolet detection wavelength was 254 nm, and the mobile phase was acetonitrile-1.0 g/L potassium dihydrogen phosphate solution at a flow rate of 1.0 mL/min. Gradient elution was adopted, the peak of the cyclohexylamine derivative appeared at a retention time of 17.75 min, and the peak area response value was the largest. The methodological validation analysis showed that the detection limit of cyclohexylamine was 0.5 mg/kg, the quantification limit was 2.0 mg/kg, and the spiked recoveries were in the range of 99.37–110.16%. The relative standard deviations (RSDs) were in the range of 0.17–1.26%. Four samples were tested and analyzed by the established method, and cyclohexylamine was not detected.

## 1. Introduction

Sodium cyclamate is a common sweetener that is 30–40 times sweeter than sucrose [1,2]. Sodium cyclamate was first approved for consumption in the United States in 1949, during which time it was considered safe and was later used in the soft drink industry beginning in the 1950s [3]. However, in 1966, it was shown that sodium cyclamate was metabolized into cyclohexylamine by intestinal bacteria. In 1969, the US National Scientific Research Council received experimental data on bladder cancer in rats fed long-term high doses of saccharin and cyclamate mixtures. In 1970, the US Food and Drug Administration (FDA) issued a ban on the use of sodium cyclamate [4]. Collings, Alan J [5] showed that experimental animals metabolized sweeteners to cycloheximide and showed that cycloheximide caused testicular atrophy in experimental animals, which raised concerns about the safety of sweeteners. Renwick, AG [6] concluded that sodium cyclamate was metabolized into cyclohexylamine in the body during long-term administration. In China, excessive intake of sodium cyclamate greatly damages human health, especially in those with weak metabolic detoxification ability, the elderly, and pregnant women, and it may even lead to cancer or birth defects. Because the safety of sodium cyclamate cannot be guaranteed, many countries have completely banned its use, and China has also implemented strict measures to ensure the safety of food processing [7–11]. Sodium cyclamate is currently synthesized by reacting cyclohexylamine with sulfamic acid and sodium hydroxide. Because cyclohexylamine does not have a conjugated group, it does not absorb ultraviolet light and cannot be detected. Therefore, dansulfonyl chloride was chosen as a derivatization reagent for cyclohexylamine to qualitatively analyze cyclohexylamine, the decomposition product of sodium cyclamate during deep-frying.

Cyclohexylamine can present acute toxicity risks. The main methods for determining cyclohexylamine include gas chromatography [12,13],ion chromatography [14], ultra-high-performance liquid chromatography-mass spectrometry [15], high-performance liquid chromatography [16], and spectrophotometry [17]. There are few reports of sodium cyclamate being fried at high temperatures, and the safety of sodium cyclamate is still debated.

In this paper, we study the changes of sodium cyclamate during high-temperature frying and establish a method to detect and quantify its main decomposition product, cyclohexylamine.

## 2 Materials and methods

### 2.1 Reagents and instruments

#### 2.1.1 Materials and reagents

Materials: Sunflower seeds, almonds, fried peanuts, chestnuts, and corn oil were purchased at a market. Reagents: Sodium cyclamate standard solution (1 mg/mL) was from ANPEL Laboratory Technologies, Inc. (Shanghai). Cyclohexylamine standard (purity > 99%) was purchased from Beijing Spectrum Analytics Standard Technology, Co. Potassium dihydrogen phosphate and sodium hydroxide (analytical purity) were obtained from Sinopharm Chemical Reagent Co., Ltd. Dansyl chloride (purity 98%) was purchased from Shanghai Yuanye Bio-Technology Co., Ltd. Acetonitrile and methanol (chromatographically pure) were purchased from Sigma-Aldrich.

#### 2.1.2 Instruments

A high-performance liquid phase chromatograph equipped with a UV Detector (Agilent1260) was used in this paper.

### 2.2 Solution preparation

Sodium cyclamate standard solution (1.0 mg/mL): Accurately pipette 1.0 mL sodium cyclamate liquid standard to a volumetric flask and increase the volume to 10 mL.

Cyclohexylamine standard reserve solution (1.0 mg/mL): Accurately pipette 10 mg of cyclohexylamine liquid standard to bring the volume up to 10 mL.

Potassium dihydrogen phosphate solution (1.0 g/L): Weigh 2.0 g of potassium dihydrogen phosphate solid, add ultrapure water to dissolve it, and then increase the volume to 2.0 L. Adjust the pH to 7 using 200 g/L potassium hydroxide solution.

Sodium hydroxide solution (50 g/L): Weigh 50 g of sodium hydroxide solid and dissolve it to make a constant volume of 1 L.

### 2.3 Liquid chromatography

On an Agilent 1260 high-performance liquid chromatograph (HPLC), the model of the column was Eclipse XDB-C18, 5 μm 4.6 × 150 mm, and the mobile phase was acetonitrile 1.0 g/L potassium dihydrogen phosphate solution with gradient elution. The column temperature was 25°C, the flow rate was 1.0 mL/min, and the injection was 5 μL.

### 2.4 Sodium cyclamate frying experiment

Take 5.0 mL of oil matrix in a test tube, add 1.2 mg of sodium cyclamate standard, and mix well. The test tubes were placed in an oil bath and pretreated at different frying temperatures (160°C, 170°C, 180°C, 190°C, and 200°C) and different frying times (5 min, 10 min, 15 min, 20 min, and 25 min). The products of sodium cyclamate standard after frying in the test tube were rinsed into a 50 mL centrifuge tube with 24 mL of ultrapure water, followed by the addition of 1 mL of potassium ferricyanide solution (150 mg/mL) and 1 mL of zinc sulfate solution (300 mg/mL). Then, the volume was brought to 50 mL by adding n-hexane. The above samples were centrifuged at 4000 rpm for 5 min, and then 10 mL of the lower solution was transferred to a 50 mL centrifuge tube. 2.0 mL of sulfuric acid (50 mL:50 mL), 5.0 mL of n-heptane, and 1.0 mL of sodium hypochlorite (50 mg/mL) were added. A vortex shaker was used to oscillate the sample for 1 min, then 25 mL of sodium bicarbonate (50 mg/mL) was added to the n-heptane layer, and the sample was oscillated for 1 min. Then, it was allowed to settle, and the organic phase was sampled. The decomposition products of sodium cyclamate standard after frying were pretreated according to GB5009.97 (China), and the filtrate was prepared for sampling.

### 2.5 Experimental methods

#### 2.5.1 Derivatization method

**Fig 1:**
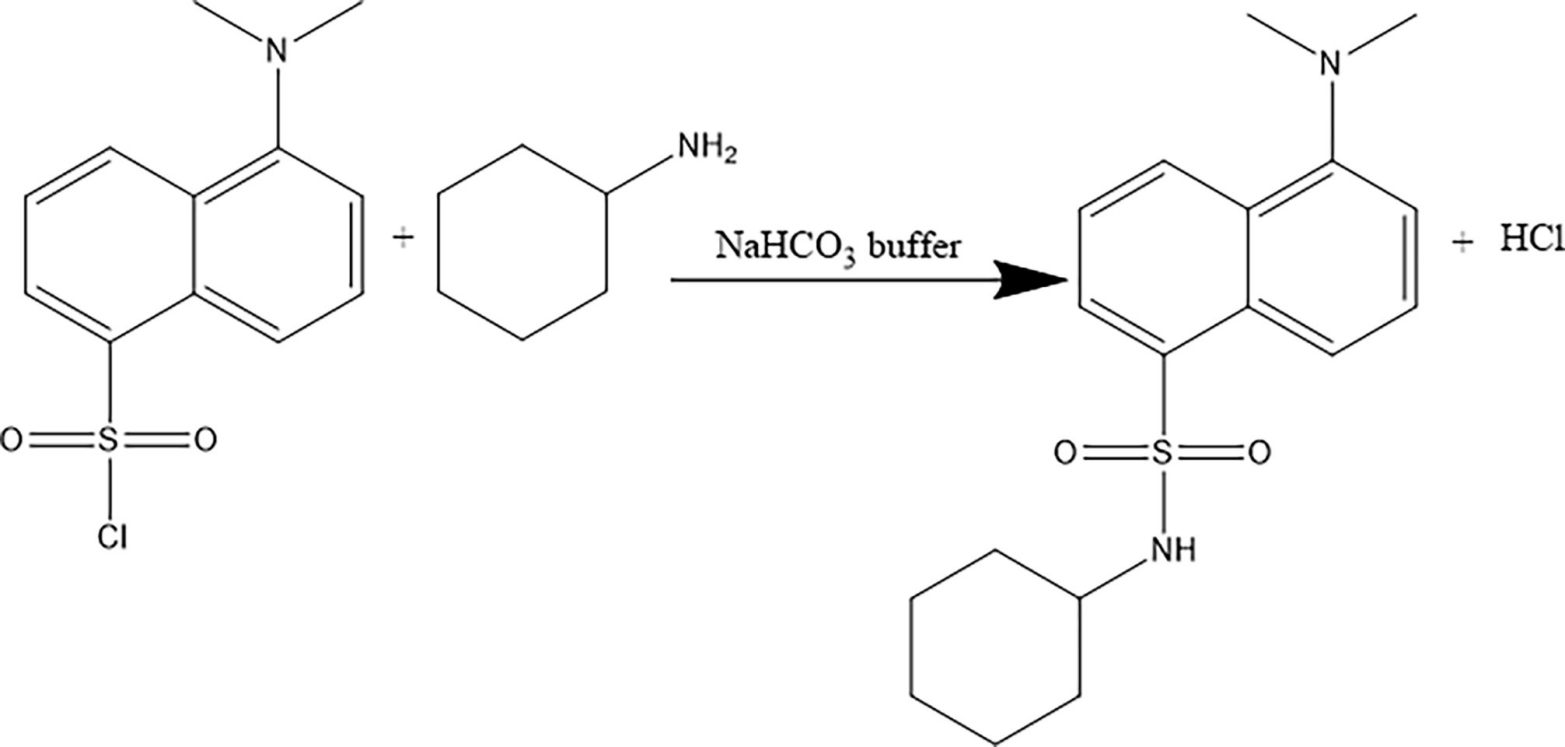
Principle of cyclohexylamine derivatization.

Cyclohexylamine can be derivatized with dansulfonyl chloride, and the derivatives can absorb UV light. In this derivatization method, dansulfonyl chloride, which has a reactive chloride group, replaces a hydrogen atom on the amino group in the cyclohexylamine molecule to produce dansulfonylcyclohexylamine and hydrogen chloride (Fig 1).

After the frying product (cyclohexylamine) was cleaned with a total of 5 mL ultrapure water three times, it was mixed three times with a cleaning solution. 1 mL of the cleaned liquid phase was placed in a 15 mL centrifuge tube, and then 1 mL of sodium bicarbonate buffer and 1 mL of dansulfonyl chloride solution were added, shaken, and then put into a 60°C water bath for 15 min for the derivatization reaction [18].

#### 2.5.2 Derivatization conditions

The derivatization experiments were optimized with four variables. The high-performance liquid-phase response values of cyclohexylamine derivatives were investigated by varying the buffer solution pH to 9.5, 10, 10.5, 11, and 11.5. The derivatization time was varied from 1 min, 10 min, 15 min, 20 min, to 25 min. The concentration of the derivatization reagent dansyl chloride was varied 0.5, 1.0, 1.5, 2.0, and 2.5 mg/mL. The derivatization reaction was carried out at 30°C, 40°C, 50°C, 60°C, and 70°C, and then determined and analyzed by HPLC-UV.

#### 2.5.3 Sample preparation

Sodium cyclamate standard reserve solution (0.5 mL) was pipetted into a glass tube, and then 1.0 mL of corn oil was added and fried at 200°C for 25 min. When the sample cooled to room temperature, the oil phase in the glass tube was washed with 5.0 mL of ultrapure water three times. Then, the aqueous phases were combined in a glass tube and vibrated until homogeneous to prepare a sample (SS-0).

Sample (SS-0; 1.0 mL) was pipetted into a glass tube, to which 1.0 mL of sodium bicarbonate buffer reserve solution and 1.0 mL of dansyl chloride solution reserve solution were added, fully shaken, and put into a 60°C water bath for derivatization. Sample (SS-1) was obtained after 15 min.

The control sample (CS-1) was prepared by pipetting 1.0 mL of corn oil into a glass tube at 200°C for 25 min. The prepared CS-1 was derivatized according to section 2.4.1 to prepare a control sample (CS-2). Pipette 1.0 mL of sodium cyclamate standard solution into a glass tube, and then perform the derivatization procedure in section 2.4.1 to obtain a control sample (CS-3). Pipette 0.5 mL of sample SS-0 into a 15 mL centrifuge tube, and add 100 μg/mL of cyclohexylamine 0.5 mL. Then, perform derivatization according to the method in section 2.4.1 to prepare a control sample (CS-4).

### 2.6 Standard curve establishment

Pipette 0.0 mL, 0.1 mL, 0.2 mL, 0.5 mL, and 1.0 mL of cyclohexylamine standard solution, and carry out the derivatization reaction according to the method in section 2.4.1. The standard curve was plotted using the concentration of cyclohexylamine (μg/mL) as the horizontal coordinate and the peak areas of the corresponding derivatives as the vertical coordinate.

## 3. Results and discussion

### 3.1 Stabilization of sodium cyclamate after frying

#### 3.1.1 Oils as substrate

**Fig 2:**
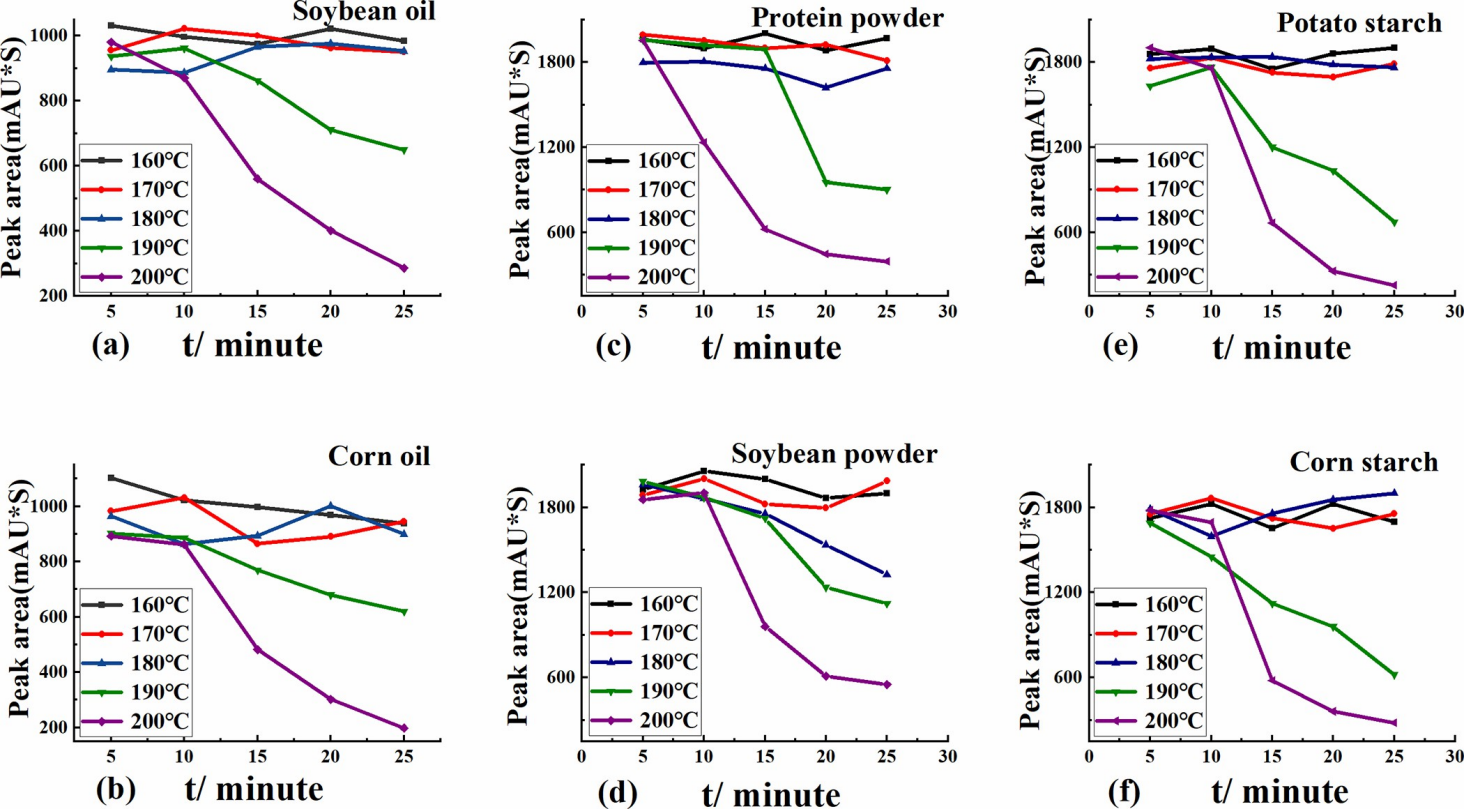
(a) Soybean oil as the matrix, (b) corn oil as the matrix, (c) protein powder as the matrix, (d) soybean powder as the matrix, (e) potato starch as the matrix, and (f) corn starch as the matrix.

**Fig 3:**
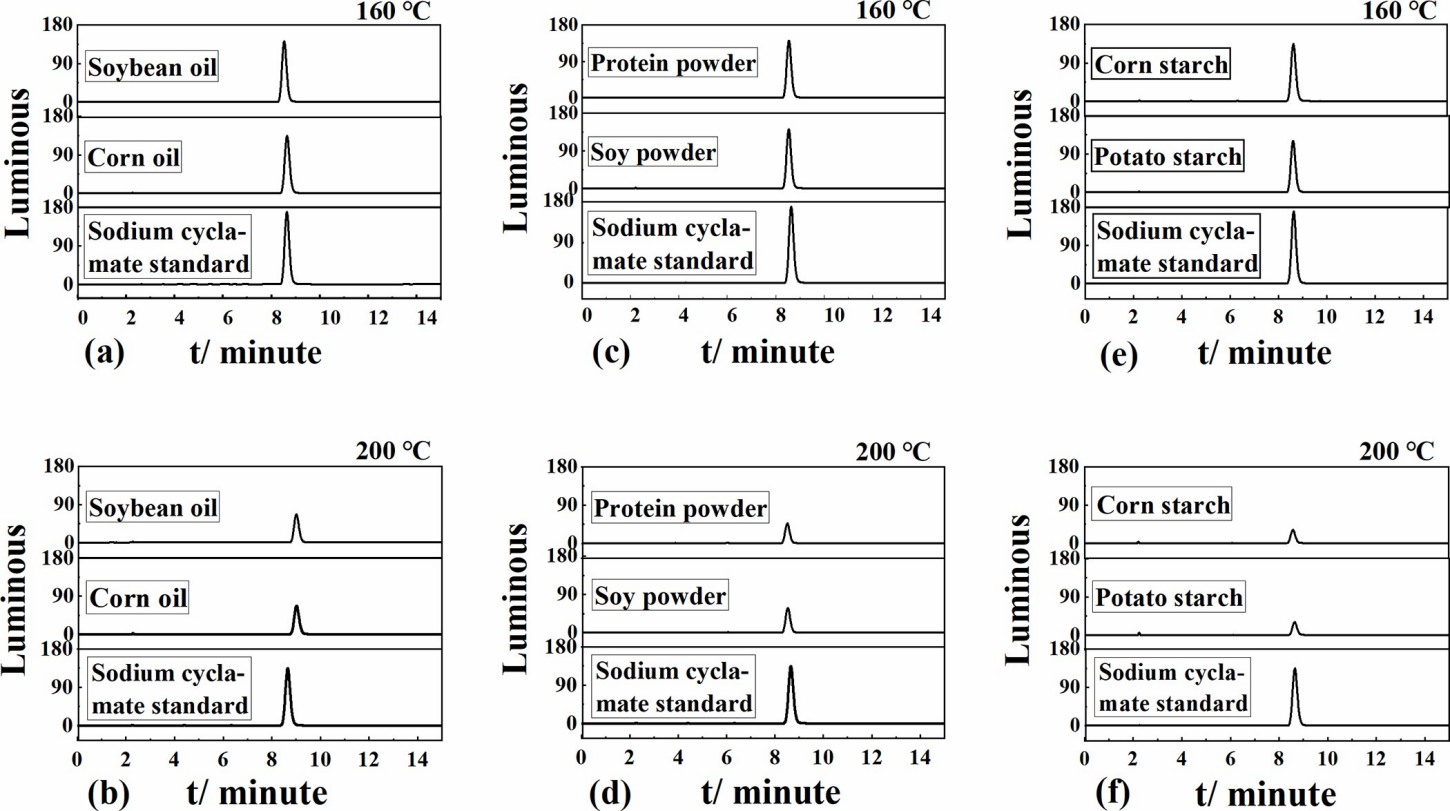
(a), (c), (e) Chromatograms at a frying temperature of 160°C for 25 min, and (b),(d),(f) chromatograms at a frying temperature of 200°C for 25 min.

Soybean oil and corn oil were used as the bases, respectively. The sodium cyclamate standard was processed according to the method in section 2.3, and the results are shown in Fig 2A and 2B. Upon increasing the frying time within the deep-frying temperature range of 160–180°C, changes in the content of sodium cyclamate standard were not significant, indicating that sodium cyclamate decomposition did not occur below 180°C. When the frying temperature exceeded 190°C, upon increasing the frying time, the content of sodium cyclamate began to decrease. After frying at 200°C for 10 min, the content of sodium cyclamate significantly changed. As shown in Fig 3A and 3B, the response values of the sodium cyclamate did not change significantly after treatment under two different conditions (Condition I: frying at 160°C for 25 min; Condition II: frying at 200°C for 25 min). The response values of the sodium cyclamate standard did not change significantly in the two oils under either condition. Comparing condition I and condition II, the response value of the sodium cyclamate standard changed very significantly, and the content of the sodium cyclamate standard was reduced a lot under condition II.

#### 3.1.2 Proteins as substrate

Changes in the sodium cyclamate standard under different frying temperatures and frying times were investigated using protein powder and soybean flour as the bases (Fig 2C and 2D). Upon prolonging the frying time at frying temperatures of 160°C, 170°C, and 180°C, the changes in the peak area of the sodium cyclamate standard were small, indicating that the sodium cyclamate standard did not significantly decompose below 180°C. When the temperature reached 190°C, the content of sodium cyclamate standard decreased upon prolonging the frying time. Obvious changes occurred at 10 min, and the content of sodium cyclamate standard began to decrease. When the frying temperature was 200°C, changes in the sodium cyclamate standard were more obvious. Fig 3C and 3D show that upon frying under both conditions, the content of sodium cyclamate standard significantly decreased, both in the protein powder matrix and soybean flour as a matrix.

#### 3.1.3 Starch as substrate

Using starch as the base, the results were similar to those obtained using protein as the base (Figs 2E, 2F, 3E and 3F).

Through the above analysis, the conditions for sodium cyclamate stabilization were determined. Sodium cyclamate remained stable when the frying temperature was in the range of 160–185°C, and the frying time was 5–25 min, and also at frying temperatures of 185–200°C and frying times of 5–8 min. Changes in the sodium cyclamate occurred when deep frying at 200°C for 25 min.

The current method to synthesize sodium cyclamate involves the reaction of cyclohexylamine with sulfamic acid and sodium hydroxide. Because cyclohexylamine does not have a conjugated group, it does not absorb UV light. Therefore, dansulfonyl chloride was chosen as a derivatization reagent to react with cyclohexylamine to qualitatively analyze cyclohexylamine, the decomposition product of sodium cyclamate after deep-frying.

### 3.2 Detection wavelength

**Fig 4:**
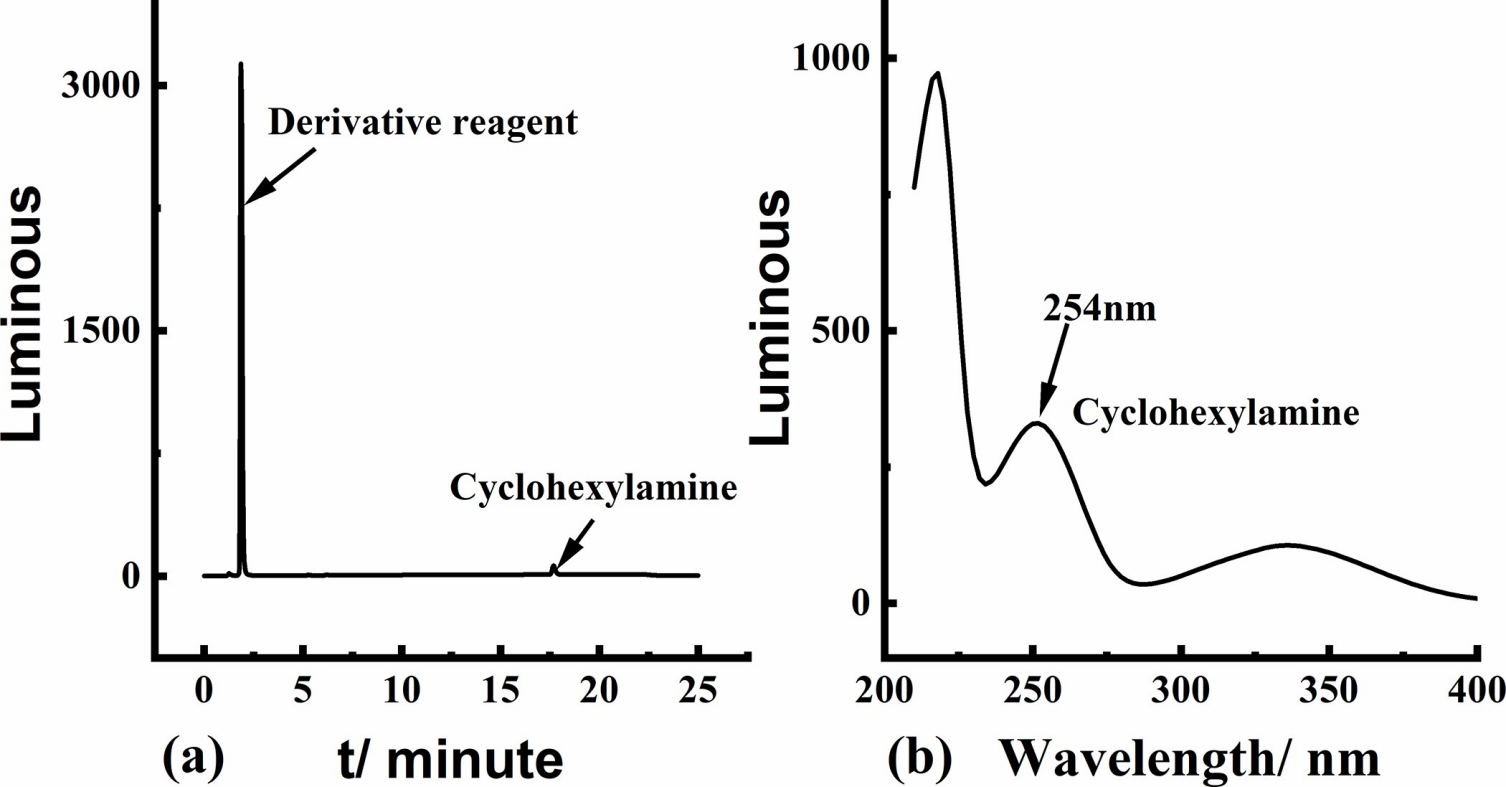
(a) Target peak chromatogram and (b) UV spectrum of cyclohexylamine.

The optimal detection wavelength for HPLC-UV was determined to be 254 nm, and the chromatogram showed a distinct peak at 17.75 min in Fig 4A and 4B.

### 3.3 Standard curve establishment

**Fig 5:**
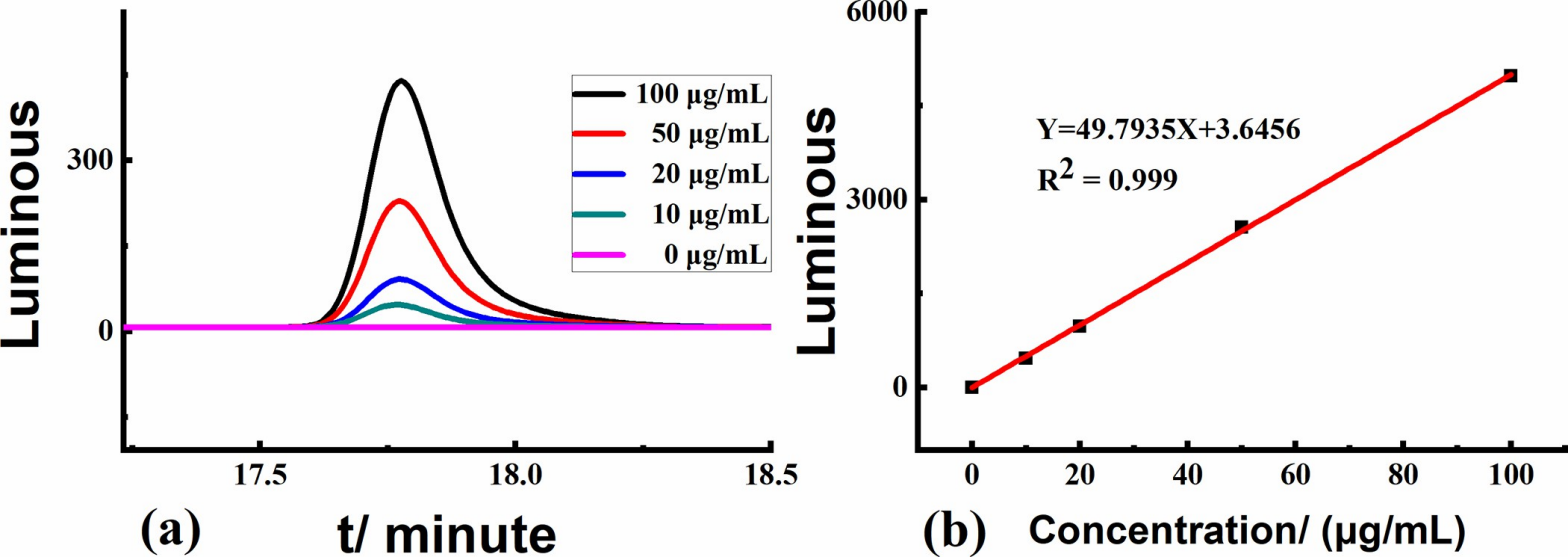
(a) Chromatogram of cyclohexylamine standard solution, and (b) cyclohexylamine standard curve.

The cyclohexylamine standard was processed according to section 2.4.1, and the resulting chromatogram is shown in Fig 5A, and the standard curve is shown in Fig 5B. The peak areas of cyclohexylamine derivatives in the range of 0–100 μg/mL showed a good linear relationship with the concentrations of the corresponding standard solutions (*Y* = 49.7935*X* + 3.6456, *R*^2^ = 0.999).

### 3.4 Chromatography

#### 3.4.1 Selection of mobile phase system

In terms of organic phases, methanol and acetonitrile were examined separately, and the elution ability of acetonitrile was stronger than that of methanol. Acetonitrile separated better than the other organic relative derivatives, with less baseline noise. The effect of water and potassium dihydrogen phosphate solution was also examined. The addition of a small amount of potassium dihydrogen phosphate improved the peak shape. Based on the target peak, the mobile phase was finalized as acetonitrile-1.0 g/L potassium dihydrogen phosphate.

#### 3.4.2 Selection of the elution method

Gradient elution was adopted using organic phase acetonitrile and inorganic phase 1.0 g/L potassium dihydrogen phosphate solution as the mobile phase. The optimal gradient elution ratio was determined to separate the cyclohexylamine derivatives (Table 1).

**Table 1:** Gradient elution schedule.

Elution time/ min	Mobile phase A /(Acetonitrile)	Mobile phase B /(1.0g/L Potassium dihydrogen phosphate)
0	30%	70%
15	70%	30%
20	70%	30%
20.1	30%	70%
25	30%	70%

#### 3.4.3 Mobile phase flow rate selection

The experiments were conducted to compare and analyze the separation of the target peaks at flow rates of 0.8, 1.0, and 1.2 mL/min, respectively. It was appropriate that the mobile phase flow rate was 1.0 mL/min.

### 3.5 Derivatization conditions

**Fig 6:**
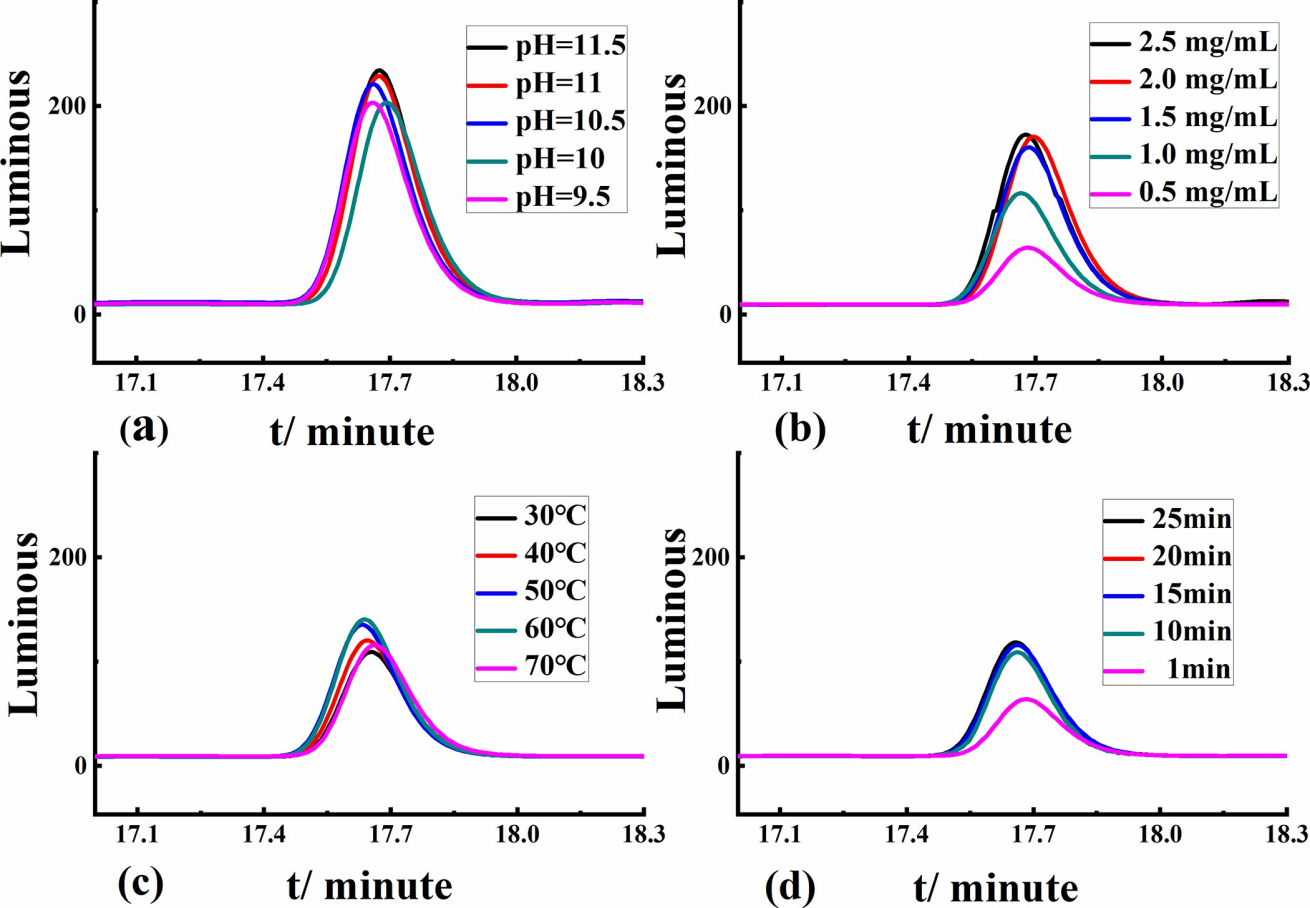
Effect of sodium cyclamate decomposition on (a) pH of buffer solution, (b) dansyl chloride concentration, (c) derivation temperature, and (d) derivation reaction time.

After the single-factor experiments, the pH of the system was adjusted by sodium bicarbonate buffer solution at pH 11 to improve the efficiency of the reaction (Fig 6A). The concentration of the derivatization reagent was selected as 2 mg/mL (Fig 6B). The results in Fig 6C show that when the temperature reached 60°C, the derivative efficiency was more significant than at other temperatures. Fig 6D shows that the derivative time peaked at 20 min, and the response value of the derivatized compounds remained unchanged.

### 3.6 Limit of detection and limit of quantification

The limit of detection (LOD) was calculated to be 0.5 mg/kg based on a 3-fold signal-to-noise ratio (*S*/*N* = 3), and the limit of quantification (LOQ) was calculated to be 2.0 mg/kg based on *S*/*N* = 10, indicating that the sensitivity of the method is acceptable.

### 3.7 Sample spiked recovery

According to the method in section 2.4.1, sodium cyclamate was used as the matrix for the spiked recovery test. Three mass concentrations of cyclohexylamine standard solutions were spiked in the range of 10–100 μg/mL, and the spiked amounts were 10 μg/g, 20 μg/g, and 100 μg/g, respectively. Each sample was prepared in parallel six times for determination. Calculations were carried out using the spiked recovery formula (Eq (1)), and the relative standard deviation formula (Eq (2)). In Eq 1, *P* denotes the spiked recovery, and *C*_*1*_ represents the total mass of cyclohexylamine when the standard solution of cyclohexylamine was added to the product of sodium cyclamate after frying. *C*_*2*_ refers to the mass of cyclohexylamine of sodium cyclamate after frying. The spiked recoveries in the range of 99.37–110.16% were obtained. The relative standard deviations were in the range of 0.17–1.26%, which met the requirements of the experimental analytical methods Table 2, indicating that the method has good precision and high accuracy.


$$P=\frac{c_{1}-c_{2}}{c_{3}}\times 100\%$$ (1)

$$RSD=\frac{SD}{X}\times 100\%$$ (2)

**Table 2:** Spiked recovery and precision of cyclohexylamine.

Sample	Spiked Levels/ (mg/kg)		Detection value/ (mg/kg)						Background	Recovery	RSD
			1	2	3	4	5	6	/(mg/kg)	/ %	/ %
Sodium cyclamate	10	Cyclohexy -lamine	9.862	10.265	9.768	9.942	9.766	10.023	0	99.37	0.17
	20		21.567	21.668	21.321	21.586	22.031	21.997	0	108.48	1.26
	100		110.235	108.961	111.257	109.523	109.958	111.002	0	110.16	0.72

### 3.8 Determination of cyclohexylamine

The content of sodium cyclamate decreased to varying degrees upon increasing the frying temperature and frying time, indicating that a decomposition reaction occurred. Thus, it was concluded that a frying temperature of 200°C and frying time of 25 min resulted in the greatest reduction in the amount of sodium cyclamate. Therefore, these conditions were used to test the pretreatment condition to stabilize sodium cyclamate. Because cyclohexylamine is a required reactant in the synthesis of sodium cyclamate, if the mass of sodium cyclamate decreases during high-temperature frying, this may indicate sodium cyclamate decomposition into cyclohexylamine. Therefore, a validation test was carried out along this line of thinking.

**Fig 7:**
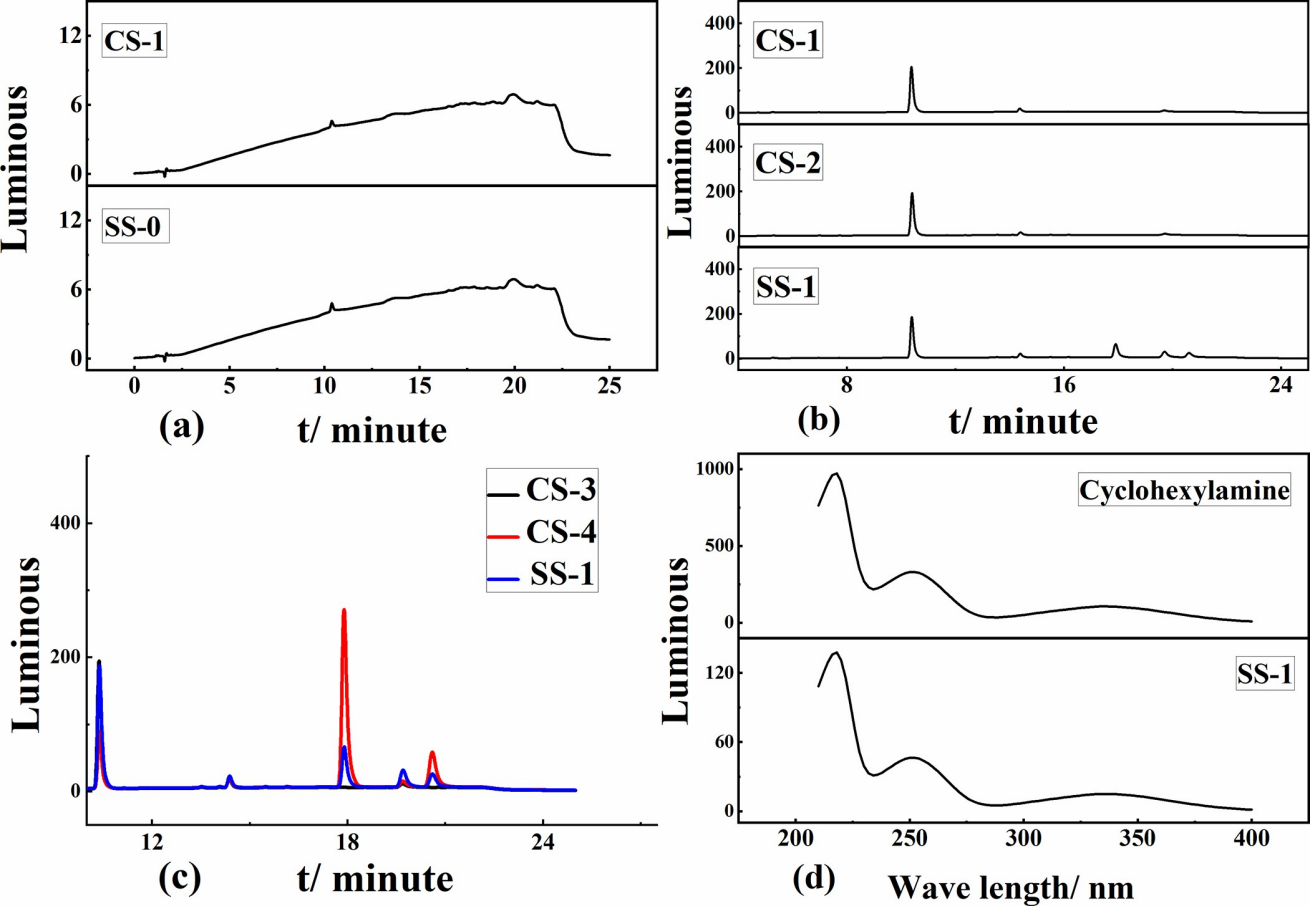
(a) Chromatogram of cyclohexylamine standard solution, (b) chromatogram of cyclohexylamine standard, (c) sample spiked chromatography, and (d) actual sample spectrogram.

Because cyclohexylamine does not have a conjugated functional group, it does not absorb in the UV region and cannot be detected by UV fluorescence. Dansyl chloride was chosen as a derivatization reagent to react with cyclohexylamine to characterize cyclohexylamine, a decomposition product of sodium cyclamate after deep-frying. Fig 7A shows that the liquid chromatograms of SS-0 and CS-1 were identical, indicating no UV-absorbing compound in the sodium cyclamate product after deep-frying. Therefore, sodium cyclamate was derivatized to render it UV-absorbing for detection by HPLC-UV. Fig 7B shows that at a retention time of 17.75 min, there was no UV absorption peak in the chromatograms of CS-1 and CS-2, indicating that CS-1 and CS-2 did not react with dansyl chloride. In contrast, there was a distinct peak in the chromatogram of SS-1, which was the product of SS-0 derivatized by dansyl chloride and could be detected by the UV detector. Therefore, frying oil and sodium cyclamate did not interfere. The next step was to determine the decomposition products of sodium cyclamate after frying. At 17.75 min, CS-3 had no UV absorption peak, and CS-4 and SS-1 had obvious peaks, and the two target peaks corresponded to the same substance (Fig 7C). Comparing the target peaks in the chromatograms of cyclohexylamine and sample SS-1 (Fig 7D) shows that these two UV absorption peaks were the same, indicating that cyclohexylamine was present in the sodium cyclamate residue after frying at 200°C for 25 min.

### 3.9 Stability tests

The stability tests were performed to examine the stability of the derivatives. Due to the complexity of the substances in the tested food samples, to prevent interference with analysis and ensure the stability of the samples, the standard solutions of sodium cyclamate and cyclohexylamine were examined separately according to section 2.4.1. The derivatized sample solutions were stored at 4°C, protected from light, and the samples were injected and measured at intervals of 1 h to investigate the stability. The obtained data were analyzed, and it was shown (Table 3) that the derivatized compounds remained stable for 10 h. The difference between the maximum and minimum concentrations was less than 1.277%.

**Table 3:** The change of front area within 10 h.

Time/ h	1	2	3	4	5	6	7	8	9	10	RSD / %
Sodium cyclamate	101.48	99.83	101.24	102.43	99.6	101.45	98.97	101.33	102.04	100.68	0.905
Cyclohexylamine	102.74	101.23	100.39	101.3	102.46	101.26	100.02	101.23	101.89	100.55	1.013
Sunflower seeds	12.4	11.93	12.43	11.38	13.13	12.61	11.86	12.06	11.63	12.11	1.215
Almond	10.3	10.93	11.38	11.02	10.22	9.98	10.91	11.06	10.4	10.21	1.064
Chetnuts	13.1	13.93	12.03	13.11	12.12	13.08	12.91	13.46	11.96	12.04	1.277
Fried peanuts	11.7	9.93	10.43	11.4	11.52	11.21	9.91	9.96	10.83	10.04	1.061

### 3.10 Actual sample testing

**Fig 8:**
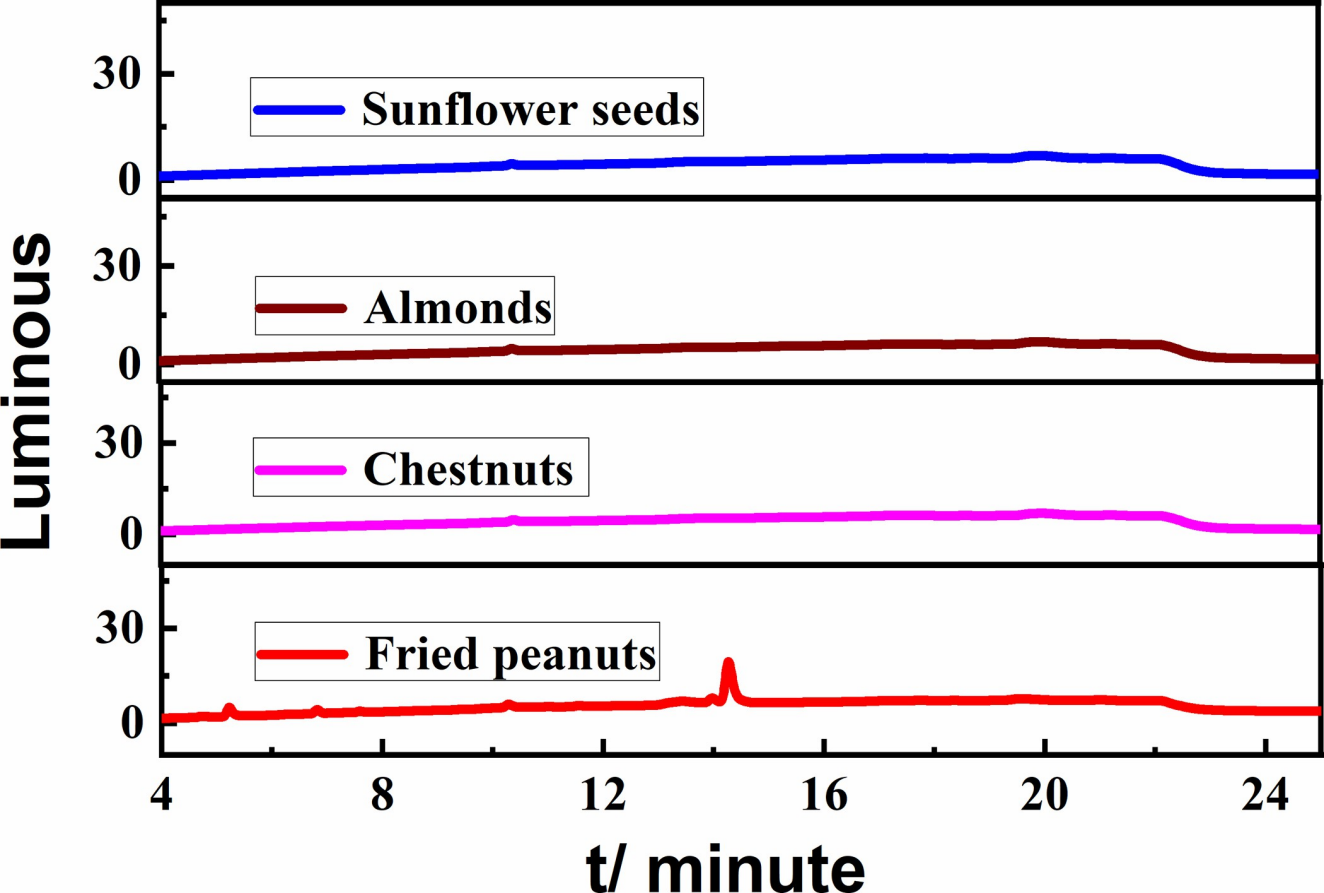
Chromatograms of the actual samples.

A total of four samples of commercially available sunflower seeds, almonds, chestnuts, and fried peanuts from different manufacturers were pre-treated. The samples were then analyzed according to the newly developed pre-column derivatization/HPLC method to determine cyclohexylamine, and the chromatograms of the samples are shown in Fig 8. Cyclohexylamine was not detected in any of the four samples.

## 4. Conclusions

Consumers are concerned about the nutritional value of food, as well as food safety hazards. Sodium cyclamate is an artificial sweetener added to foods that may thermally decompose into toxic compounds during frying, so the application of sweeteners in fried foods should be limited. The results revealed that sodium cyclamate remained stable within the range of 160–185°C for 5–25 min and at 185–200°C for 5–8 min. When deep-frying at 200°C for 25 min, the sodium cyclamate content greatly decreased, and cyclohexylamine was produced. A pre-column derivatization/HPLC method for the determination of cyclohexylamine was established and showed good precision and high accuracy. Four samples of commercially available nuts and seeds were analyzed by the established method, and cyclohexylamine was not detected.

## Supporting information

S1 File (XLSX)
